# Growth rates of human induced pluripotent stem cells and neural stem cells from attention-deficit hyperactivity disorder patients: a preliminary study

**DOI:** 10.1007/s00702-023-02600-1

**Published:** 2023-02-17

**Authors:** Cristine Marie Yde Ohki, Natalie Monet Walter, Audrey Bender, Michelle Rickli, Sina Ruhstaller, Susanne Walitza, Edna Grünblatt

**Affiliations:** 1 Department of Child and Adolescent Psychiatry and Psychotherapy, Psychiatric University Hospital Zurich, University of Zurich, Zurich, Switzerland; 2 Neuroscience Center Zurich, University of Zurich and the ETH Zurich, Zurich, Switzerland; 3 Zurich Center for Integrative Human Physiology, University of Zurich, Zurich, Switzerland; 4 Biomedicine PhD Program, University of Zurich, Zurich, Switzerland; 5 Department of Child and Adolescent Psychiatry and Psychotherapy, Translational Molecular Psychiatry, Psychiatric University Hospital Zurich, University Zurich, University of Zurich, Wagistrasse 12, 8952 Schlieren, Switzerland

**Keywords:** ADHD, iPSC, Neural stem cell, Growth, Proliferation, WST-1, xCELLigence, Real-time cellular impedance monitoring

## Abstract

Attention-deficit hyperactivity disorder (ADHD) is a neurodevelopmental polygenic disorder that affects more than 5% of children and adolescents around the world. Genetic and environmental factors play important roles in ADHD etiology, which leads to a wide range of clinical outcomes and biological phenotypes across the population. Brain maturation delays of a 4-year lag are commonly found in patients, when compared to controls of the same age. Possible differences in cellular growth rates might reflect the clinical observations in ADHD patients. However, the cellular mechanisms are still not elucidated. To test this hypothesis, we analysed the proliferation of induced pluripotent stem cells (iPSCs) and neural stem cells (NSCs) derived from male children and adolescents diagnosed with ADHD and with genetic predisposition to it (assessed using polygenic risk scores), as well as their respective matched controls. In the current pilot study, it was noticeable that NSCs from the ADHD group proliferate less than controls, while no differences were seen at the iPSC developmental stage. Our results from two distinct proliferation methods indicate that the functional and structural delays found in patients might be associated with these in vitro phenotypic differences, but start at a distinct neurodevelopmental stage. These findings are the first ones in the field of disease modelling of ADHD and might be crucial to better understand the pathophysiology of this disorder.

**Supplementary Information:**

The online version contains supplementary material available at 10.1007/s00702-023-02600-1.

## Introduction

Attention-deficit hyperactivity disorder (ADHD) is one of the most common neurodevelopmental disorders with a worldwide prevalence of over 5% affecting children and adolescents, from which ca. 60% of the cases persist into adulthood (Sharma and Couture 2014; Banaschewski et al. 2017). Clinicians estimate brain maturational delays of about 3 to 5 years, with ADHD patients displaying difficulties in behaviour control and attentive behaviour (Posner et al. 2020; Shaw et al. 2007). These symptoms elicit challenges in everyday life in terms of academic performance and social function (Abrahão and Elias 2021). While the etiology is still not fully understood, multiple genetic factors have been discovered to be strongly associated with ADHD (Palladino et al. 2019; Wood and Neale 2010). Genome wide association studies (GWAS) have revealed risk *loci* in ADHD, involving genes encoded for brain-related processes such as synapse formation, neuronal development, and plasticity, indicating maturational processes being affected (Demontis et al. 2019). Moreover, studies report abnormalities in brain structure and delayed maturational processes in regions associated with cognitive processes in ADHD patients (Hoogman et al. 2019; Shaw et al. 2007). In consistency to these findings, meta-analysis of gene variants displays negative correlations of intracranial volume, showing a significant overlap between genetics and brain volume measures in ADHD (Klein et al. 2019). ADHD is considered a multifactorial disorder, demonstrating phenotypic and genetic variability, signifying the need for personalized research (Franke et al. 2009; Koi 2021). Therefore, it is of importance to recreate the disorder in an accurate model that recapitulates the genetic background of individuals (Green et al. 2022; Faraone and Larsson 2019). Induced pluripotent stem cell (iPSC) technology displays a relevant tissue model, as cell reprogramming can be conducted on non-invasively obtained somatic cells from patients (Wiegand and Banerjee 2019; Yde Ohki et al. 2022). Human iPSCs allows the research of different developmental stages from iPSCs to mature neurons, mimicking embryonic neurogenesis (Compagnucci et al. 2014).

Neurogenesis is a complex process mediating stem cell proliferation and differentiation relevant for expansion and cellular specification of the brain, from which a tightly regulated timeline plays a critical role (Andrews and Nowakowski 2019). During healthy brain development, neural stem cells (NSCs) are driven to coordinate the interplay between self-renewal, further differentiation to intermediate progenitor cells, and direct differentiation to neurons (Guillemot 2005). Furthermore, NSCs display an important intermediate for the rapid expansion of the brain during neurogenesis (Andrews and Nowakowski 2019; Rajan et al. 2021). An imbalance in early brain development can lead to detrimental consequences for individuals causing neurodevelopmental disorders, such as schizophrenia, autism spectrum disorders (ASD) and ADHD (Courchesne et al. 2020; Friedman and Rapoport 2015; Pantelis et al. 2005).

The current study aims to elucidate neural development in ADHD by evaluating proliferation rates in iPSCs and iPSC-derived NSCs, representing important developmental stages throughout embryonic neurogenesis. Our hypothesis encompasses the proliferation rate being attenuated in ADHD indicating developmental delays as suggested by previous publications (Berger et al. 2013; Dark et al. 2018). To assess the proliferation in iPSCs and NSCs, impedance-based analysis using the xCELLigence settings, with measurements taken throughout 5 days, was considered (Grünblatt et al. 2018). To validate these results, further growth rate analysis using WST-1 assays for 4 days was taken into account. These findings will support previous assumptions of developmental delays at a cellular level and furthermore allow a fundamental approach to further investigate potential treatments in ADHD.

## Methods

### Recruitment of participants

Children and adolescents with ADHD (responding to MPH treatment) and healthy controls (from 6 to 18 years old) were recruited by the Department of Child and Adolescent Psychiatry and Psychotherapy (KJPP) of the University of Zurich (UZH). Inclusion and exclusion criteria were described in previous publications of our group (Grossmann et al. 2021; Yde Ohki et al. 2021). Details about the participants involved in this study (3 ADHD patients *versus* 3 healthy controls) can be found in Supplementary Table 1.

After recruitment, salivary DNA samples from patients and controls were submitted to genotyping, as described in previous publications (Grossmann et al. 2021; Yde Ohki et al. 2021). Individual polygenic risk scores (PRS) were calculated as an indicator of genetic predisposition to ADHD, using the software PLINK 1.9 (Chang et al. 2015). Individual PRS were computed using the PRS-auto method with EUR super-population from 1000 Genomes as the linkage disequilibrium reference panel and based on the genome-wide significant risk loci data from Demontis et al. (Demontis et al. 2019; Ge et al. 2019).

### Generation and culture of iPSCs

Plucked hair-derived keratinocytes and peripheral mononuclear blood cells (PBMCs) from ADHD patients and healthy controls were collected and reprogrammed into iPSCs using Sendai virus transduction. Quality control (QC) from generated iPSCs was performed as reported in previous publications (Grossmann et al. 2021; Yde Ohki et al. 2021). The QC of iPSC lines K015 c1 and c9, which have not been included in these previous reports, is shown in Supplementary Fig. 1.

### Quality control of iPSC-derived NSCs

When NSCs reached p5, NSCs underwent gene and protein expression analysis through RT-qPCR and immunocytochemistry assays, respectively. In the former, the expression of NSC genes *PAX6*, *SOX2* and *NES* (Nestin) was investigated, using *ACTB* and *C5orf18* (REEP5) as reference genes. The technical procedure was performed as described in Grossmann et al. and Yde Ohki et al., and the primers used are available in Supplementary Table 2 (Grossmann et al. 2021; Yde Ohki et al. 2021).

For immunocytochemistry, protein expression of the classical markers FOXG1, NESTIN, SOX2 and TUJ1 was observed (Supplementary Fig. 2). Details about the primary and secondary antibodies used in this protocol may be found in Supplementary Table 3.

Once the NSCs reached around 90–100% of confluence in 96 wells, the media was removed, and the cells were carefully washed with PBS 1X. Paraformaldehyde (PFA) at 4% was added and cells were incubated for 20 min at room temperature (RT). After incubation, PFA was aspirated properly discarded in special waste and the wells were rinsed 3 times with PBS 1X for 5 min each. After the last washing step, blocking buffer (0.1% Triton^™^ X-100, 1% BSA in PBS) was added to the wells, which were incubated for 30 min at RT.

In the meantime, the primary antibody mixtures were prepared in blocking buffer on ice. The following antibody combinations can be considered: FOXG1/NESTIN and TUJ1/SOX2. After the incubation period, the antibody combinations were transferred accordingly to the wells, which were incubated overnight at 4 °C.

On the following day, primary antibodies were removed and the cells, washed 3 times with PBS 1X for 5 min each. Next, the NSCs were washed with blocking buffer for 5 min. A secondary antibody mixture was then prepared and added to cells, which were incubated for 30 min in the dark at RT.

To finalize the procedure, the cells were rinsed 3 times with PBS 1X for 5 min each. After the last step, one drop of fluorescence mounting medium (Agilent) was added to each well. Images were subsequently acquired using an IX81 microscope (Olympus) and the cellSens Dimension software (version 3.1) (Olympus).

### *Assessment of cell proliferation of iPSCs and NSCs *via* xCELLigence real-time impedance cell analysis*

Human iPSCs that were generated and checked for QC in our study were tested in xCELLigence. Real-time impedance-based cell proliferation of generated iPSCs and NSC was measured using the xCELLigence^®^ real-time cell analysis (RTCA) system (ACEA Biosciences).

Each iPSC line was seeded in 12 replicates onto E-96 plates (OLS^®^ BIO) coated with Vitronectin 5 μg/mL (Gibco^™^) beforehand and stored overnight at 4 °C. Baseline was measured during 1 min after temperature equilibration with Essential 8^™^ Flex medium (E8 Flex, Gibco^™^) for 30 min at 37 °C and 5% CO_2_. iPSCs were counted using Countess^™^ II FL Automated Cell Counter (Thermo Fisher Scientific^™^) and seeded at a concentration of 25′000 cells/well. For NSCs, 15′000 cells/well were seeded onto Geltrex (Gibco^™^) -coated E-96 plates, diluted 1:100 in DMEM/F12 (Gibco^™^), and cultivated in Neural Expansion Media (NEM; PSC Neural Induction Medium, Gibco^™^).

For both cell types, media change started  one day after seeding the cells and was performed every other day by replacing the medium with fresh E8 Flex or NEM, respectively. Cell proliferation was automatically measured every hour within 5 days. Analysis was performed using RTCA Software (version 2.0) and the graph was generated using the GraphPad Prism software (version 9.0).

Growth rate was determined according to a Malthusian growth model using a least squares method to fit the non-linear regression curve on the cell index (CI) data during the exponential growth period defined from 24 h to CImax set as the first peak followed by 5 steps decline (R Statistical Software v. 4.1.2). Outliers between the replicates were removed according to the interquartile range (IQR) method. For iPSCs, at least 9 replicates per experiment were analysed, while for NSCs, at least 3 replicates were analysed in two independent experiments each. Mean of the wells were plotted per experiment.

### *Assessment of cell proliferation of iPSCs and NSCs *via* WST-1 assays*

Using the same cell concentrations per well as in xCELLigence, 25′000 iPSCs /well were seeded onto Vitronectin-coated 96-well plates (final concentration of 5 μg/mL in PBS 1x (Gibco^™^)). On the other hand, NSCs were seeded at a final concentration of 15′000 cells/well onto Geltrex-coated 96 wells (1:100 dilution in DMEM/F12). Cells were seeded in triplicates on 8 different plates for each time point (24 h, 42 h, 48 h, 66 h, 72 h, 90 h, 96 h and 114 h) in E8 Flex or NEM. For both iPSCs and NSCs, the media were completely refreshed every other day.

At each time-point 10 µL of WST-1 tetrazolium salt (Sigma) was added in each well and the plate was incubated for 4 h in a humidified atmosphere at 37 °C, 5% CO_2_. Four hours later, the absorbance was measured for each sample using the Mithras2 LB 943 Multimode Reader from Berthold Technologies at 440 nm with a reference wavelength at 630 nm. For iPSCs, this read-out was normalized against the background control (wells without cells) considered as blank. Each experiment was performed in triplicates, which were verified for outliers at each time-point by the z-score cleaning method. For iPSCs, three independent experiments were performed, in which the slope of the log2 transformed curve generated from 24 h to maximal absorbance was considered after applying a linear regression model (R Statistical Software v. 4.1.2). For NSCs, two independent experiments were performed and growth rates were computed using a linear regression from 24 h to maximal absorbance (GraphPad Prism software (version 9.0)).

For cell viability analysis, the mean of absorbance values from 96 and 114 h post-seeding (hps) per experiment were plotted.

### Data analysis

Statistical tests and graphs were performed using the GraphPad Prism software (version 9.0). When data were normally distributed but with unequal standard deviations, two-tailed unpaired *t* tests with Welch’s correction were applied. On the other hand, two-tailed unpaired nonparametric Mann–Whitney tests were applied for data that were not normally distributed. A *p* value of at least 0.05 was considered significant.

## Results

**Fig. 1:**
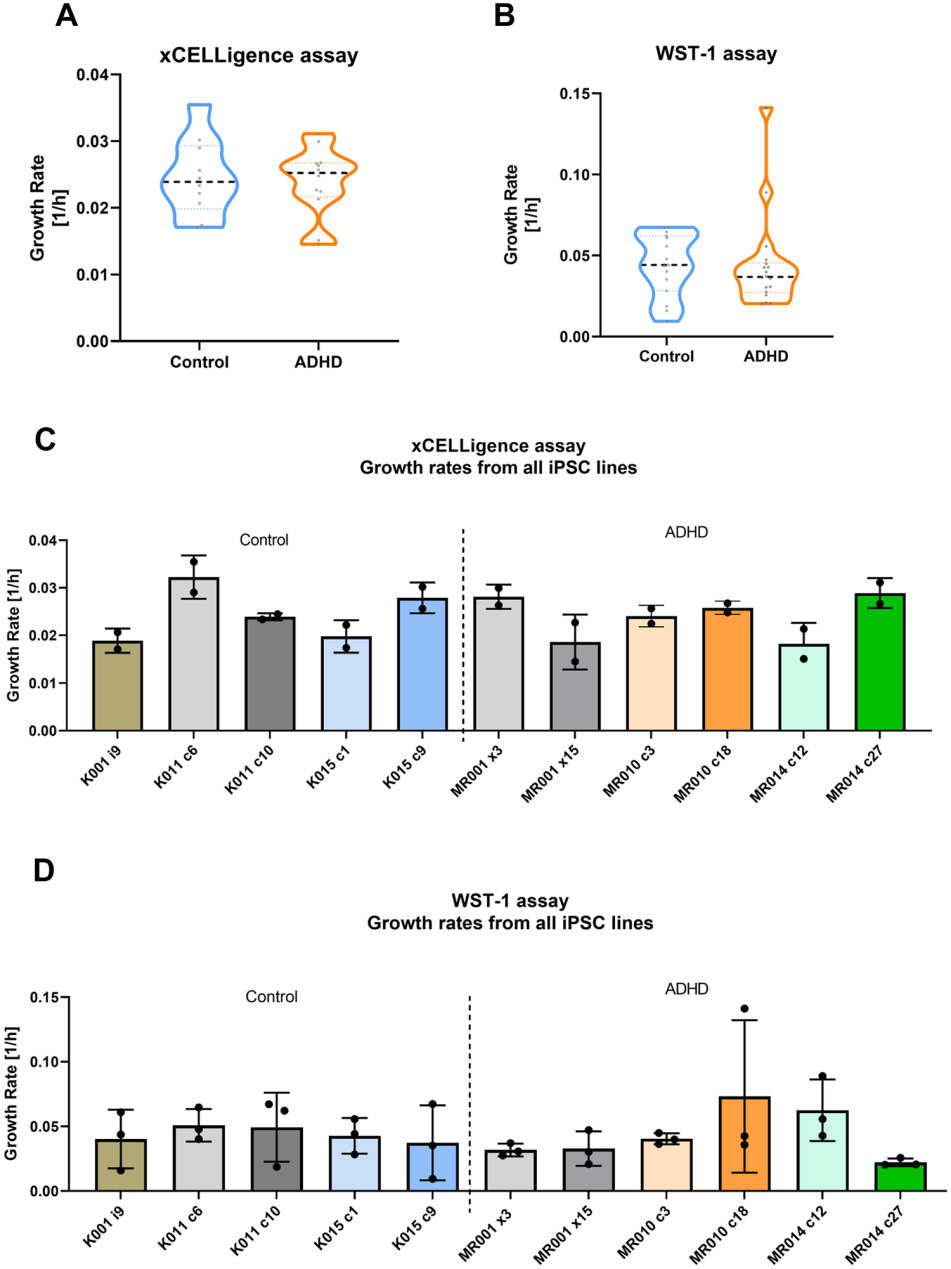
Growth rate analysis by xCELLigence and WST-1 assays in iPSCs. **A** Real time impedance-based results from xCELLigence show that no significant differences were seen between proliferation rates of both groups. **B** WST-1 results in iPSCs show the same pattern of response as xCELLigence. For xCELLigence: two-tailed unpaired *t* test with Welch’s correction; n.s. for iPSCs (*n* = 5/3 control and *n* = 6/3 ADHD lines/individuals, 2 independent experiments). For WST-1: two-tailed unpaired nonparametric Mann–Whitney test, n.s. (*n* = 5/3 control and *n* = 6/3 ADHD lines/individuals in 3 independent experiments). Growth rates from individual cell lines in xCELLigence (**C**) and WST-1 assays (**D**) are depicted

**Fig. 2:**
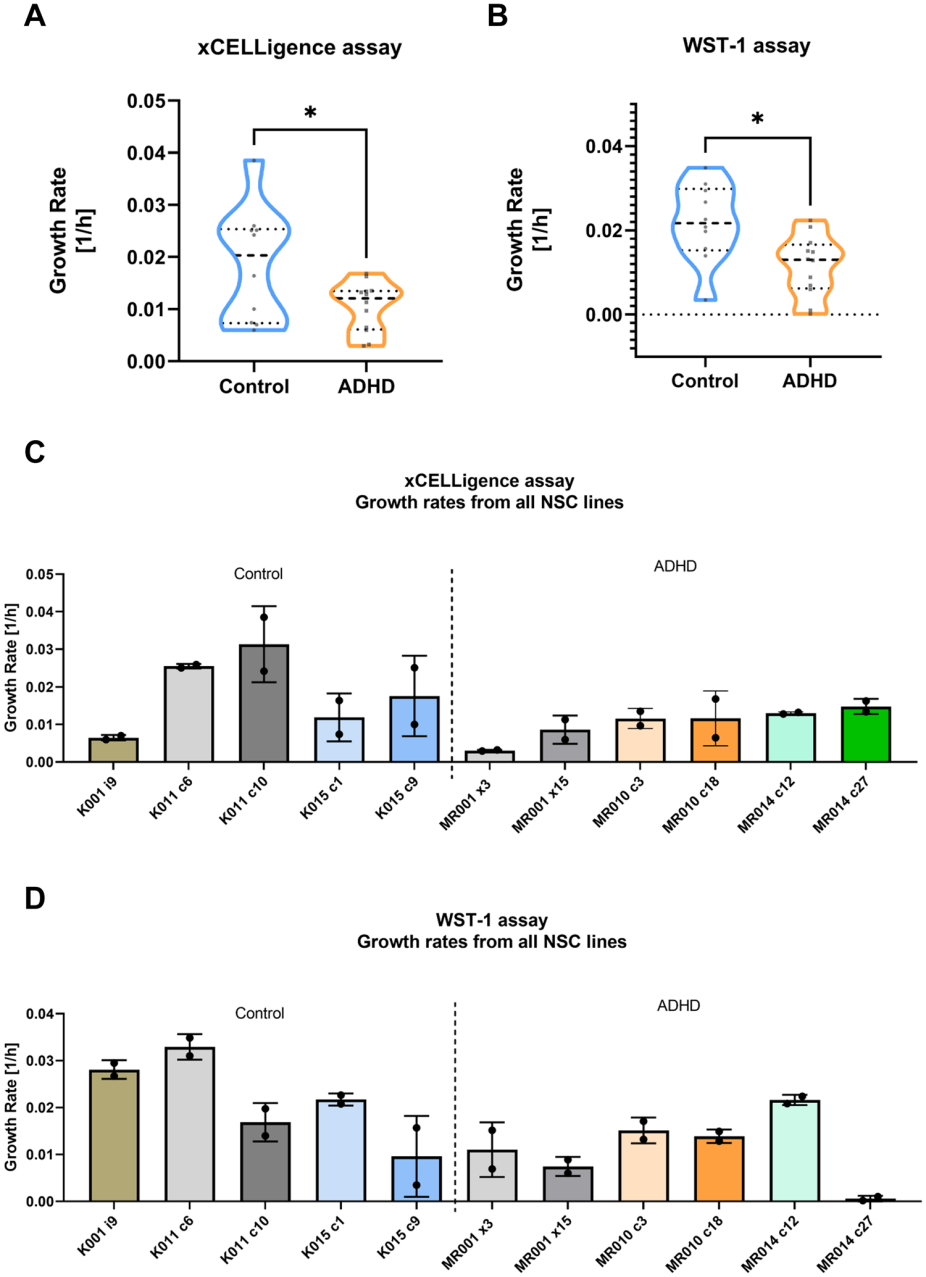
Growth rate analysis by xCELLigence and WST-1 assays in NSCs. Growth rates are significantly higher in controls compared to the ADHD group in xCELLigence (**A**) and WST-1 (**B**) assays. For xCELLigence: two-tailed unpaired *t* test with Welch’s correction, *p = 0.0495 (*n* = 5/3 control and *n* = 6/3 ADHD lines/individuals in 3 independent experiments). For WST-1: two-tailed unpaired *t* test with Welch’s correction, *p = 0.0113 (*n* = 5/3 control and *n* = 6/3 ADHD lines/individuals in 2 independent experiments). Growth rates from individual cell lines in xCELLigence (**C**) and WST-1 assays (**D**) are depicted

In the current pilot study, we investigated only cell lines derived from males, in which the patients were those with positive PRS z-score for ADHD, while the controls had negative PRS for ADHD genetic predisposition (See Supplementary Table 1). Individual growth rates for each iPSC line are depicted in Fig. 1C, D, while Fig. 2C, D depicts individual NSCs.

In the results for iPSC proliferation, we observed no significant differences in growth rates between ADHD and control groups measured using the xCELLigence (Fig. 1A; two-tailed unpaired *t* test with Welch’s correction, *t*(18.23) = 0.2514, *p* = 0.8043), while WST-1 assays have confirmed the non-significant finding (Fig. 1B; Mann–Whitney *U* = 110, *p* = 0.3807). On the other hand, growth rates from ADHD NSCs were significantly lower than controls in the real-time impedance method xCELLigence (Fig. 2A; two-tailed unpaired *t* test with Welch’s correction, *t*(11.88) = 2.187, *p* = 0.0495), as well as for the WST-1 method (Fig. 2B; two-tailed unpaired *t* test with Welch’s correction, *t*(16.74) = 2.846, *p* = 0.0113).

**Fig. 3:**
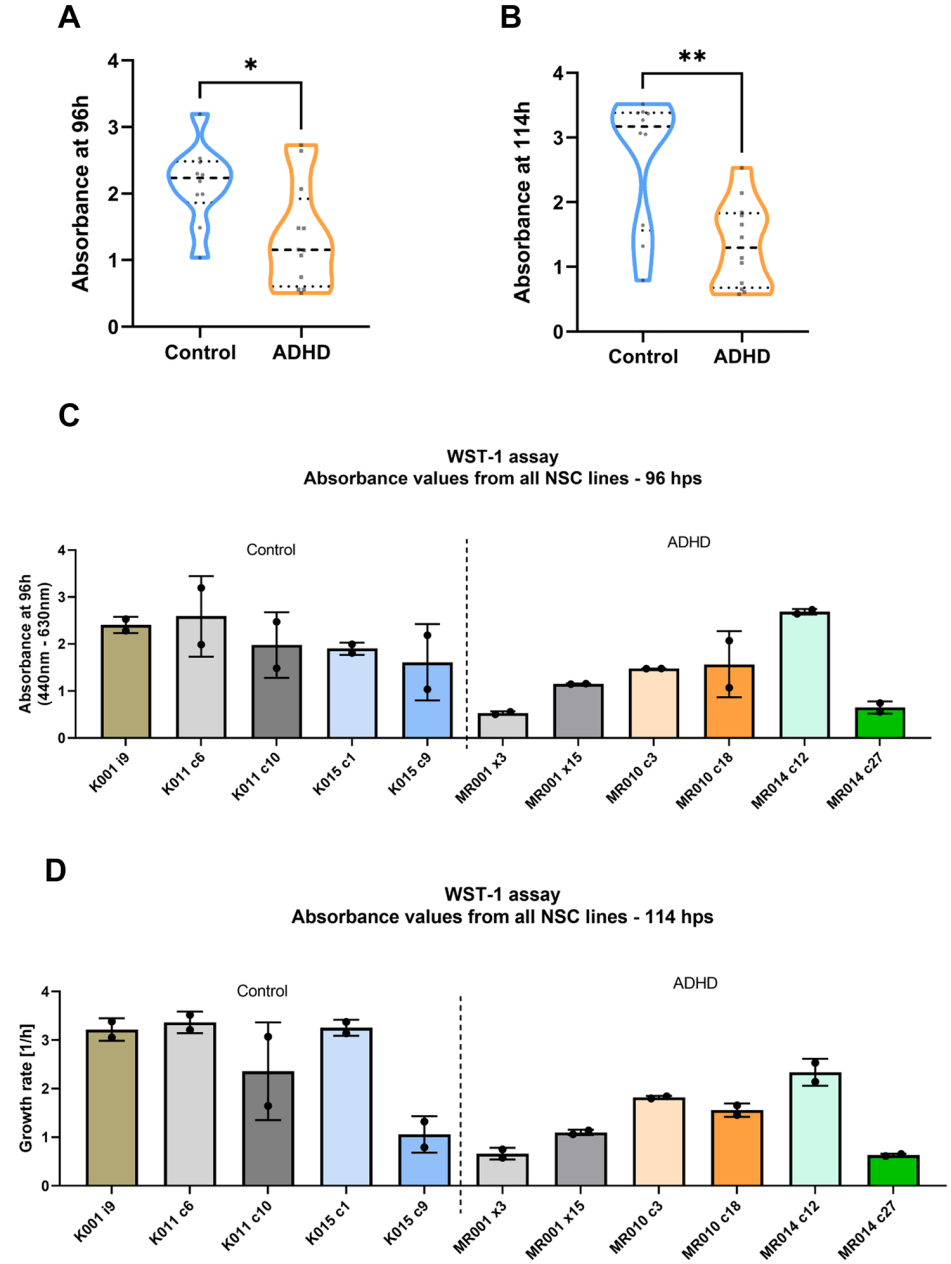
Cell metabolism analysis from NSCs at 96 (**A**) and 114 h post-seeding (**B**). Results were obtained from WST-1 assays. As statistical tests, unpaired *t* test with Welch’s correction and Mann–Whitney were performed, respectively (*p = 0.0123 for 96 h and **p = 0.0056 for 114 h) (*n* = 5/3 control and *n* = 6/3 ADHD lines in 2 independent experiments). Absorbance values from individual cell lines at 96 h (**C**) and 114 h (**D**) are also shown

When NSC WST-1 experiments were analysed at each time-point separately, differences in the measurements were found for the latest time-points: 96 (Fig. 3A; two-tailed unpaired *t* test with Welch’s correction, *t*(19.86) = 2.755, *p* = 0.0123) and 114 h post-seeding (Fig. 3B; Mann–Whitney *U* = 19, *p* = 0.0056). At both time-points, ADHD NSCs seem to show lower metabolic rates than controls. Individual absorbance values are presented in Fig. 3C, D for 96 and 114 hps, respectively.

## Discussion

Growth rates are one of the key measurements to assess specific phenotypes at the cellular and molecular levels in several neuropsychiatric conditions. Imbalances between NSC proliferation and differentiation have been previously associated with neurodevelopmental disorders (Ernst 2016). As supporting evidence show, genes that might be related with these two cellular processes also seem to be involved in those disorders, such as *MECP2* in Rett syndrome (Li et al. 2014), *ODC1* for schizophrenia and other neuropsychiatric disorders (Prokop et al. 2021) and *UBE3A* in Angelman’s syndrome (Mishra et al. 2009). In this pilot study, we aimed to investigate differences in cell growth rates of two distinct neurodevelopmental stages: iPSCs and NSCs from ADHD patients.

Even though iPSCs of ADHD patients and controls do not display any differences in our pilot study, ADHD NSCs proliferate significantly less than controls. Additionally, the cellular metabolic rate in ADHD seems to be lower than in controls, as already seen in patients in terms of hypometabolism and structural brain maturational delays (Zametkin et al. 1990; Rubia 2007); however, this will need further confirmation using additional metabolic measures, such as mitochondria activity or LDH assays. ADHD is a disorder marked by structural and functional brain maturational delays that might include and involve decreased cortical thinning (Shaw et al. 2006; Hoogman et al. 2017). Functional connectivity and executive functioning are also found to be impaired in ADHD (Wang et al. 2020; Zhang et al. 2020). These clinical observations might be associated with the differences found in the growth of our NSCs, as they are often discussed as part of an imbalance between proliferation and differentiation through molecular mechanisms that are still unknown.

The differences between iPSC and NSC phenotypes in vitro might be attributed to the fact that dynamic changes in transcriptomic and proteomic profiles are temporarily required throughout neural differentiation (Burke et al. 2020; Varderidou-Minasian et al. 2020; Kuruş et al. 2021). These observations led us to hypothesize that the phenotypic differences in terms of growth rates tend to happen in a more advanced developmental stage.

Interestingly, the same outcome was found in another neurodevelopmental disorder: Pitt-Hopkins Syndrome (PTHS). Papes et al. have shown reduced proliferation of iPSC-derived PTHS NSCs, while no differences were seen between the proliferation of PTHS and control iPSCs (Papes et al. 2022).

In accordance, defects in NSC proliferation have been reported in other neuropsychiatric disorders. For instance, iPSC-derived neural cells from autism spectrum disorder (ASD) patients were previously assessed. In this case, NSC from patients were proliferating more than controls, which might be contributing to the macrocephaly clinically observed in ASD (Marchetto et al. 2017). On this report, the Wnt signalling pathway was pointed out as a contributor to this altered phenotype, due to its role in NSC homeostasis by regulating proliferation and differentiation (Lie et al. 2005; Marchetto et al. 2017). Considering the same rationale, multiple evidence to distinct alterations in this pathway has been linked to ADHD pathogenesis (Yde Ohki et al. 2020) and needs to be further investigated at the cellular level.

In schizophrenia, NSCs from patients have been found to proliferate less than controls (Reif et al. 2006). As reviewed by Räsänen and colleagues, the observed decreased proliferation of this cell type could be involved with a premature neuronal differentiation, which could also have been associated with the Wnt signalling (Räsänen et al. 2022).

Here, we report a preliminary novel finding that might be of interest to the ADHD field. Using iPSC-derived neural cell lines allows us to maintain the genetic background of ADHD patients, while reproducing ex vivo cellular phenotypes. Despite the validity and strong contribution of animal models to ADHD research, the iPSC technology offers an advantageous alternative to the use of these models, which would not be able to represent the human polygenic profile of ADHD and its symptoms in a high fidelity manner, as well as human commercially available neural cell lines (*e.g.*, SH-SY5Y neuroblastoma cells). However, the use of iPSC-derived 3D brain organoids would be helpful to study in the future the ADHD pathophysiology in a more complex and heterogeneous scenario.

## Limitations

Although our study is resourceful for the initial studies in ADHD disease modelling, there are some limitations i.e. the small sample size, and studying only males. However, already in this preliminary study, a significant difference in growth was observed. Nevertheless, we aim to overcome this in future studies, by adding further individuals (including females) for more gender balanced and highly powered study.

Regarding age differences, participants from ADHD and control groups were not perfectly age matched, which could be considered a limitation. Up to this date, as multiple studies have shown, the irreversibility of age-related epigenetic identity from somatic cells through the reprogramming process is still debatable, as reviewed by (Simpson et al. 2021). In our data, no correlation between the age of participants and growth rates in both methods for both cell types was observed (Supplementary Figs. 3 and 4).

Following the same rationale, differences between iPSC lines coming from distinct somatic cell types may also be considered a discussion topic (Ohi et al. 2011; Sareen et al. 2014). Our analysis have shown that no differences were seen between the proliferation of iPSCs and NSCs originating from PBMCs or keratinocytes for WST-1 and xCELLigence (Supplementary Figs. 5 and 6).

We also need to take into account that only patients with positive ADHD PRS z-scores (as well as controls with negative z-scores) are being assessed in the current preliminary study. Considering the fact that this disorder is a spectrum, patients with low genetic liability to ADHD must also be investigated, as well as respective controls with high genetic predisposition. Balancing ADHD and control groups in this context would also be essential to understand the interplay between genetic and environmental risk factors in this disorder.

Regarding techniques, here we used two different methodologies to assess proliferation. Although WST-1 assays can be performed in distinct time-points to analyse how metabolically active cells are over time based on metabolism (Scarcello et al. 2020; Ghasemi et al. 2021; Aslantürk 2018), they only reflect the metabolic rate of a cell culture and do not directly quantify its exact number of viable cells (Berridge et al. 2005), while measuring in real-time proliferation of one single well across time-points is not possible, as opposed to xCELLigence (Stefanowicz-Hajduk and Ochocka 2020). Therefore, different technical approaches result in different variabilities, in which xCELLigence gives more stable results compared to the WST-1, which needs different wells for each time-point measurements.

Colorimetric WST-1 assays are commonly used to measure cell viability, by quantifying the amount of formazan dye derived from the enzymatic cleavage of tetrazolium salts (in this case, WST-1) in viable cells, indirectly measuring mitochondrial activity (Berridge et al. 2005). Therefore, higher absorbance values indicate higher metabolic activity that could be explained by a higher number of cells or higher metabolic rates from the same amount of cells. Given that proliferation rates are significantly lower in ADHD NSCs by two different methods, the first option was to be expected. However, the second possibility cannot be completely ruled out. Thus, being aware of the technical differences, as well as the advantages and disadvantages of each method, is necessary to take into consideration.

## Conclusion

Up to this date, no studies revealing growth differences between cells derived from ADHD and healthy controls have been published. Although further investigation about the differentiation potential of NSCs would be required to elucidate possible discrepancies between ADHD and control lines, our pioneer findings already point out that imbalances in proliferation of patient-specific NSCs might be involved with brain maturational delays found in ADHD patients, in a cell-type dependent manner. To test this hypothesis, we are currently enlarging our sample size and balancing groups for gender and genetic predisposition to ADHD.

## Supplementary Information

Below is the link to the electronic supplementary material.

Supplementary file1 (PDF 1478 kb)

## Data Availability

The data presented are available upon request.
