# Supplementary material for: Growth rates of human induced pluripotent stem cells and neural stem cells from attention-deficit hyperactivity disorder patients: a preliminary study
Source: J Neural Transm (Vienna). 2023 Feb 17;130(3):243–52. doi: 10.1007/s00702-023-02600-1 (PMC10033475; doi:10.1007/s00702-023-02600-1)
Supplement: Supplementary file 1 — Supplementary file1 (PDF 1478 kb) [file 702_2023_2600_MOESM1_ESM.pdf]

Growth rates of human induced pluripotent stem cells and neural stem cells from Attention-Deficit Hyperactivity Disorder patients: a preliminary study.

Cristine Marie Yde Ohki[1,4*], Natalie Monet Walter[1*], Audrey Bender[1], Michelle Rickli[1], Sina Ruhstaller[1], Susanne Walitza[1,2,3], Edna Grünblatt[1,2,3]

[1] Department of Child and Adolescent Psychiatry and Psychotherapy, Psychiatric University Hospital Zurich, University of Zurich, Zurich, Switzerland

[2] Neuroscience Center Zurich, University of Zurich and the ETH Zurich, Zurich, Switzerland

[3] Zurich Center for Integrative Human Physiology, University of Zurich, Switzerland

[4] Biomedicine PhD Program, University of Zurich, Switzerland

*Both authors contributed equally

Supplementary Table 1. Demographic data from iPSC lines used in the pilot study.

Cell line	Diagnosis	Age	Gender	PRS (z-score)	Source of somatic cells	Clones analysed in this pilot study
K001	Healthy control	15	Male	-0.55	Plucked-hair derived keratinocytes	i9
K011	Healthy control	16	Male	-1.23	Plucked-hair derived keratinocytes	c6 and c10
K015	Healthy control	13	Male	-2.31	PBMCs	c1 and c9
MR001	ADHD	15	Male	0.13	Plucked-hair derived keratinocytes	x3 and x15
MR010	ADHD	9	Male	1.85	Plucked-hair derived keratinocytes	c3 and c18
MR014	ADHD	13	Male	1.25	PBMCs	c12 and c27

Supplementary Table 2. Primers for NSC characterization via RT-qPCR.

Target	Expected amplicon size	Reference number from the company (Qiagen)
PAX6	113bp	According to manufacturer Qiagen 249900 (QT00071169)
SOX2	64bp	According to manufacturer Qiagen 249900 (QT00237601)
NES	77bp	According to manufacturer Qiagen 249900 (QT00235781)
ACTB	146bp	According to manufacturer Qiagen 249900 (QT00095431)
C5orf18 (REEP5)	108bp	According to manufacturer Qiagen 249900 (QT00068131)

Supplementary Table 3. Antibodies for NSC characterization via immunocytochemistry.

**Primary Antibody**	**Species**	**Dilution**	**Company**	**Catalog number**
FOXG1	Rabbit	1:100	Thermo fisher	ab18259
NESTIN	Mouse	1:500	Millipore	MAB5326
TUJ1	Mouse	1:500	Abcam	ab14545
SOX2	Rabbit	1:500	Millipore	AB5603
**Secondary Antibody**	**Species made in**	**Dilution**	**Company**	**Catalog Number**
Cy™3 AffiniPure Donkey Anti-Rabbit IgG (H+L)	Donkey	1:500	Jackson immunoresearch	711-165-152
Alexa Fluor® 488 AffiniPure Donkey Anti-Mouse IgG (H+L)	Donkey	1:1000	Jackson immunoresearch	715-545-151

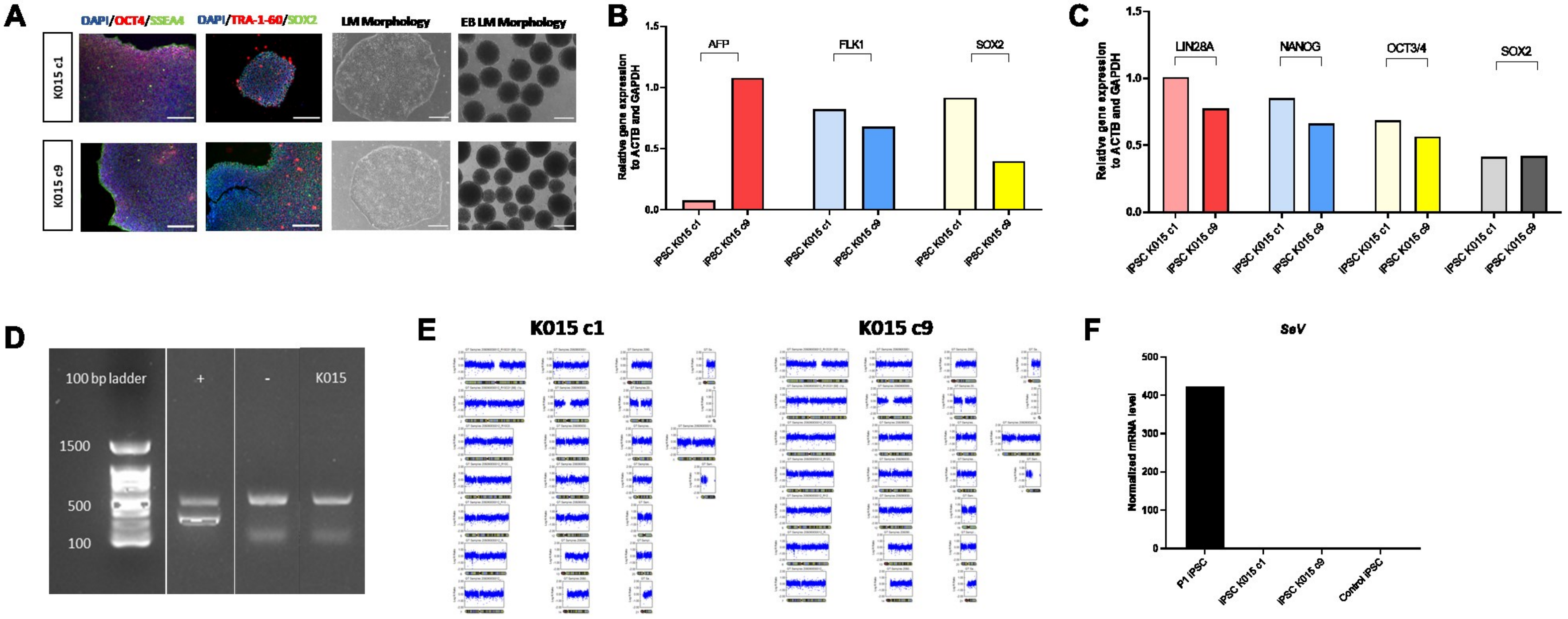

Supplementary Figure 1. Quality control of iPSC K015 c1 and c9. A) Protein expression of pluripotency markers OCT4, SSEA4, SOX2 and TRA-1-60 via immunocytochemistry, as well as phase contrast images showing classical iPSC morphology and development of embryoid bodies (EBs). Scale bar: 200 µm. B) Gene expression analysis of endodermal, mesodermal and ectodermal genes in iPSC-derived EBs (AFP, FLK1 and SOX2, respectively), through RT-qPCR. C) Pluripotency genes (LIN28A, OCT4, NANOG and SOX2) were also assessed through RT-qPCR. D) Mycoplasma testing using supernatant of iPSCs, showing no contamination from the individual K015. The gel was cropped for better appreciation of the results. E) Karyograms from iPSC K015 c1 and c9, showing no genomic aberrations after the reprogramming procedure. F) By using RT-qPCR analysis, the presence of remaining Sendai virus traces were checked, compared to the positive control iPSC p1. Only negligible expression of Sendai genome is seen.

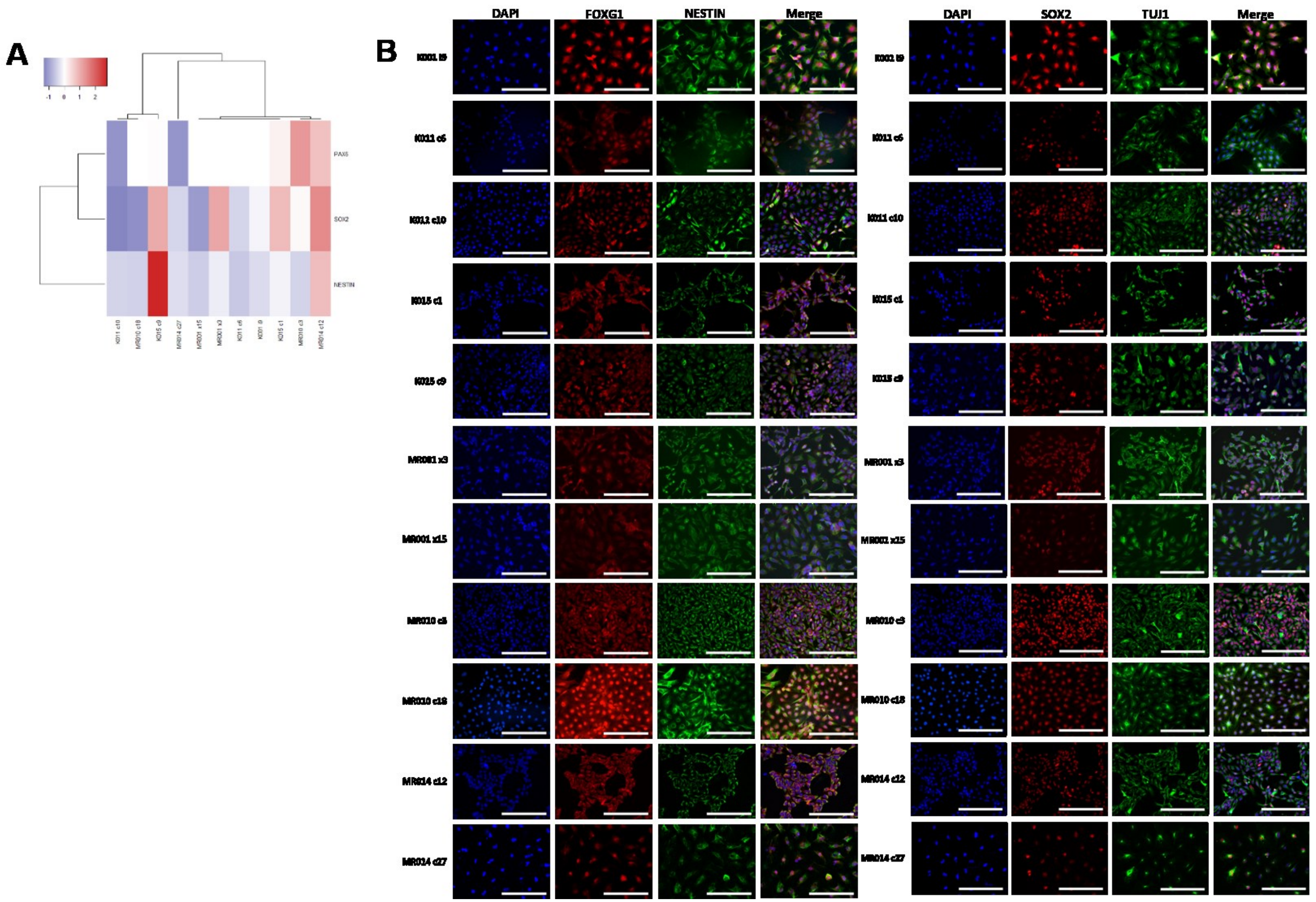

Supplementary Figure 2. Quality control of all NSCs included in this study. A) Heatmap showing gene expression of *PAX6*, *SOX2* and *NESTIN* in NSCs after RT-qPCR. B) Protein expression of the NSC markers FOXG1, NESTIN, SOX2 and TUJ1 via immunocytochemistry. Scale bar: 200 µm.

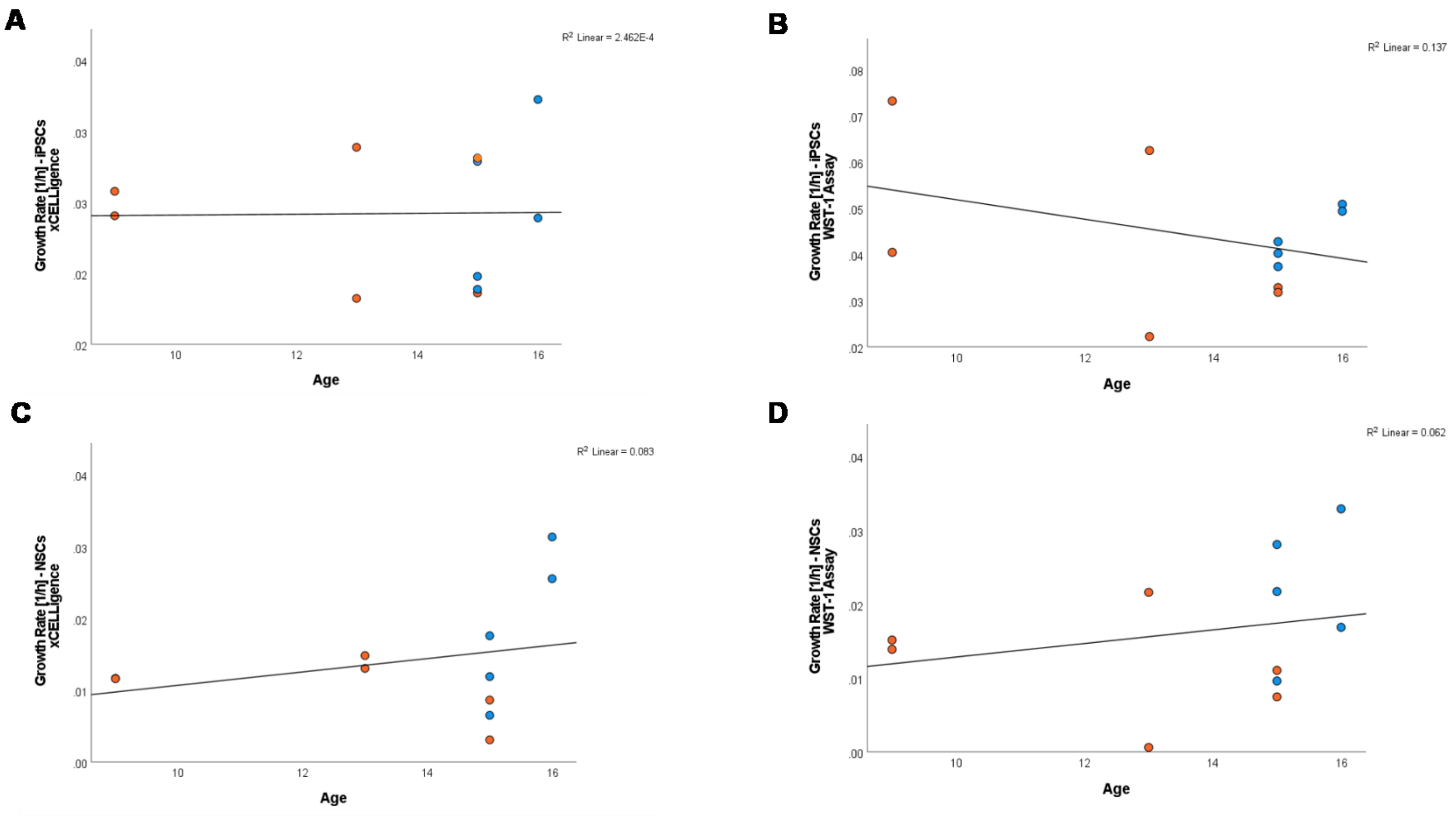

Supplementary Figure 3. Correlational analysis between individuals' age and growth rates of iPSCs and NSCs. No significant correlation was observed between growth rates of iPSCs in xCELLigence (A) and WST-1 (B), as well for NSCs in xCELLigence (C) and WST-1 assays (D). Here, the average results from the technical replicates were analyzed. Blue dots: control lines; orange dots: ADHD lines. Two-tailed nonparametric Spearman correlation tests were applied for every analysis (n.s.). For A, B, C and D, respectively: r=0.115, n=11, p=0.736; r=0.062, n=11, p=0.855; r=0.360, n=11, p=0.276; r=0.355, n=11, p=0.283.

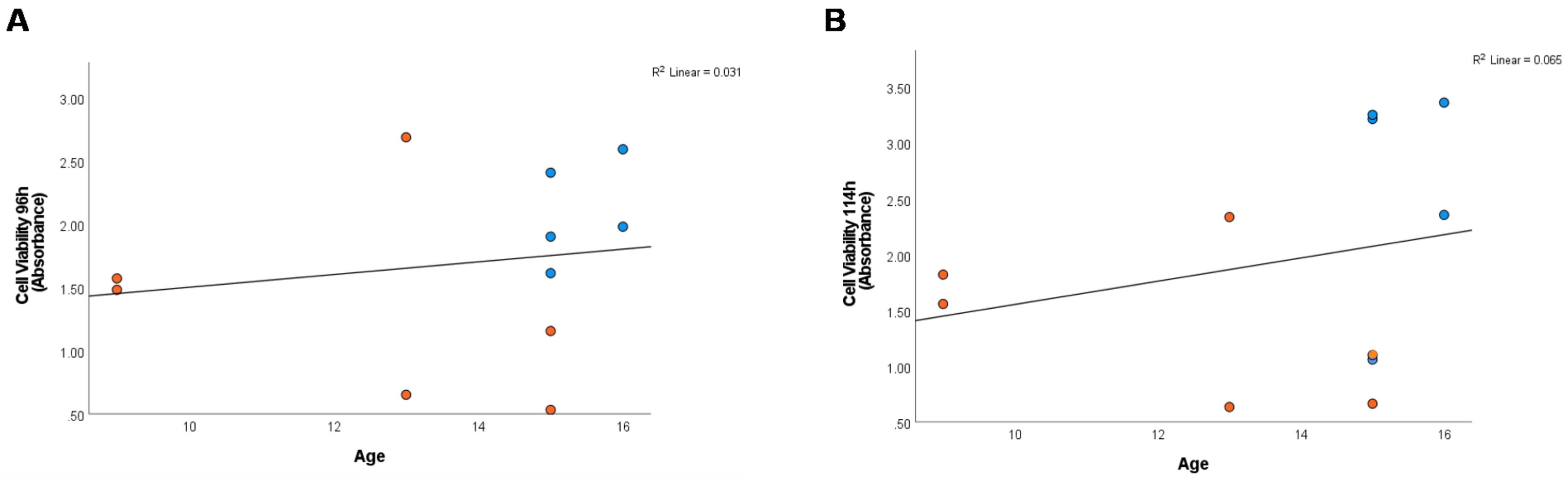

Supplementary Figure 4. Correlational analysis between individuals' age and NSC cell viability. Cell viability from NSCs at 96 hps (A) and 114 hps (B) were not significantly correlated to age of participants. Here, the average results from the technical replicates were analyzed. Blue dots: Control lines; orange dots: ADHD lines. Two-tailed nonparametric Spearman correlation tests were applied for every analysis (n.s.). For A and B, respectively: r=0.327, n=11, p=0.327; r=0.423, n=11, p=0.195.

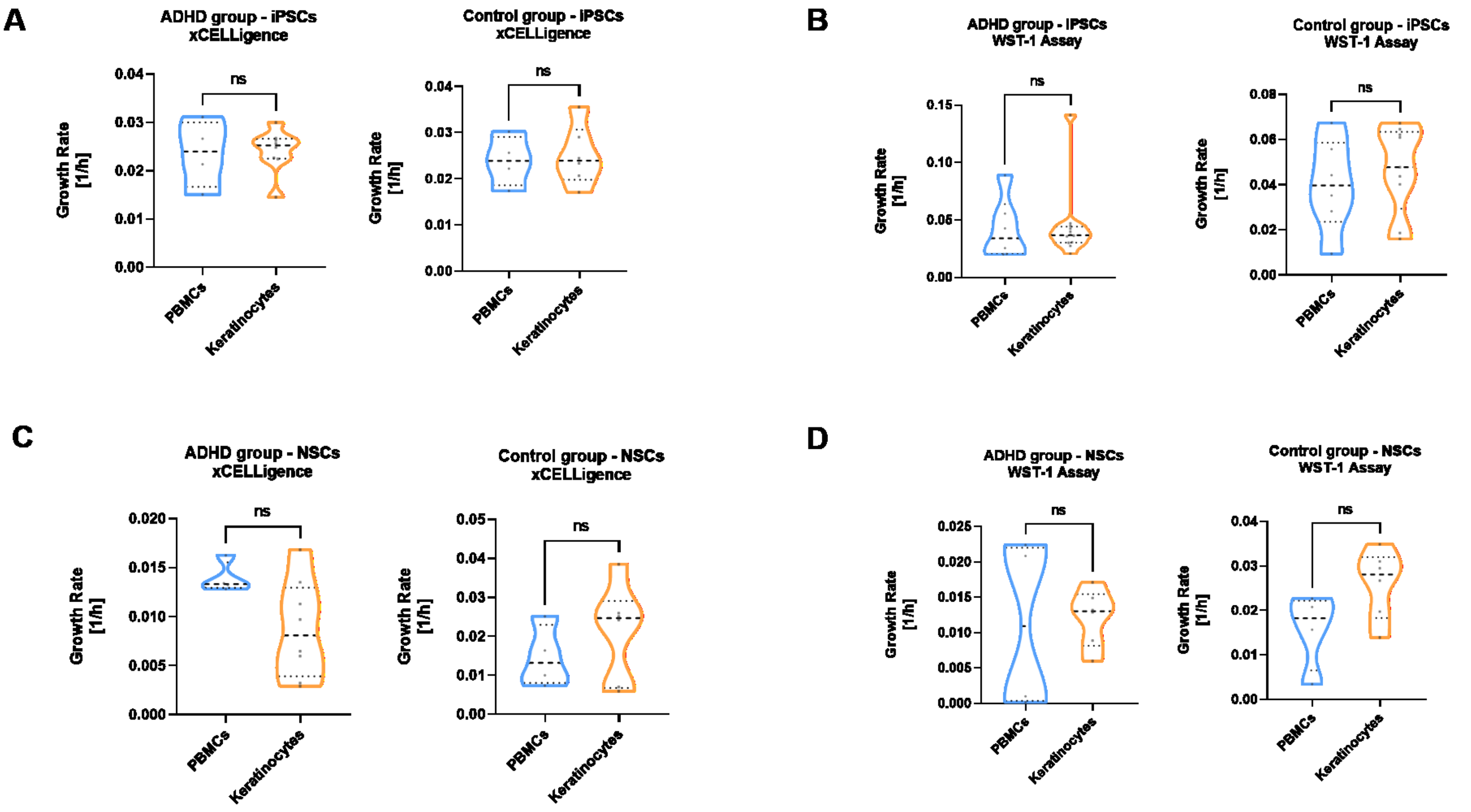

Supplementary Figure 5. Differences in group-specific growth rates between iPSCs or NSCs derived from PMBCs or hair-plucked derived keratinocytes. Comparison between iPSC growth rates by xCELLigence (A) and WST-1 assays (B) derived from PBMCs or keratinocytes. The same pattern of response is seen for NSCs in xCELLigence (C) and WST-1 (D). The ADHD group is depicted on the left panels of all items, while controls are shown on the right side. Mann Whitney tests were applied for every analysis (n.s.).

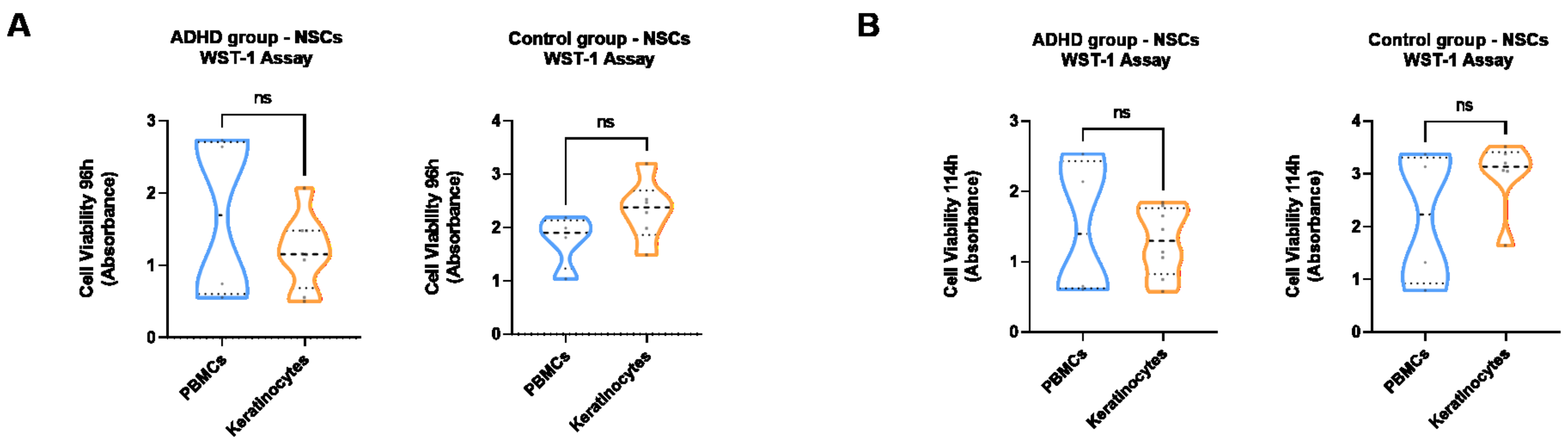

Supplementary Figure 6. Differences in group-specific cell viabilities between NSCs derived from PMBCs or hair-plucked derived keratinocytes. Cell viability from NSCs at 96 hps (A) and 114 hps (B) were compared and showed no significant differences. The ADHD group is depicted on the left panels of items A and B, while controls are shown on the right side. Mann Whitney tests were applied for every analysis (n.s.).
